# Deep Learning Ensemble Approach for Predicting Expected and Confidence Levels of Signal Phase and Timing Information at Actuated Traffic Signals

**DOI:** 10.3390/s25061664

**Published:** 2025-03-07

**Authors:** Seifeldeen Eteifa, Amr Shafik, Hoda Eldardiry, Hesham A. Rakha

**Affiliations:** 1 Charles E. Via, Jr. Department of Civil and Environmental Engineering, Virginia Tech, Blacksburg, VA 24061, USA; seteifa@vt.edu (S.E.); ashafik@vt.edu (A.S.); 2 Department of Computer Science, Virginia Tech, Blacksburg, VA 24061, USA; hdardiry@vt.edu

**Keywords:** signal phasing and timing data, traffic signal timing prediction, machine learning, ensemble machine learning techniques

## Abstract

Predicting Signal Phase and Timing (SPaT) information and confidence levels is needed to enhance Green Light Optimal Speed Advisory (GLOSA) and/or Eco-Cooperative Adaptive Cruise Control (Eco-CACC) systems. This study proposes an architecture based on transformer encoders to improve prediction performance. This architecture is combined with different deep learning methods, including Multilayer Perceptrons (MLP), Long-Short-Term Memory neural networks (LSTM), and Convolutional Long-Short-Term Memory neural networks (CNNLSTM) to form an ensemble of predictors. The ensemble is used to make data-driven predictions of SPaT information obtained from traffic signal controllers for six different intersections along the Gallows Road corridor in Virginia. The study outlines three primary tasks. Task one is predicting whether a phase would change within 20 s. Task two is predicting the exact change time within 20 s. Task three is assigning a confidence level to that prediction. The experiments show that the proposed transformer-based architecture outperforms all the previously used deep learning methods for the first two prediction tasks. Specifically, for the first task, the transformer encoder model provides an average accuracy of 96%. For task two, the transformer encoder models provided an average mean absolute error (MAE) of 1.49 s, compared to 1.63 s for other models. Consensus between models is shown to be a good leading indicator of confidence in ensemble predictions. The ensemble predictions with the highest level of consensus are within one second of the true value for 90.2% of the time as opposed to those with the lowest confidence level, which are within one second for only 68.4% of the time.

## 1. Introduction

Phasing and timing (SPaT) messages are one of the major forms of infrastructure to vehicle (I2V) communication streams that have major implications for ITS development and deployment in different states in the US. The SPaT message contains information about every phase in the traffic signal controller. For every phase, the SPaT message contains information about the status of the phase (green, red, yellow) as well as the start time, the minimum end time, and the maximum end time of each phase. The SPaT message is supposed to contain the most likely end time for the phase and the certainty in that prediction. Having a reliable stream of SPaT predictions is essential for several applications. These applications fall into two distinct categories. The first is improving signalized intersection safety, which includes turn assist, red light warning, and rail crossing red light violation [1]. The second application is concerned with improving traffic operations. This includes reducing delays in a subset of road users, such as transit signal priority, freight signal priority, and emergency vehicle preemption [1]. It also includes improving the vehicle fuel consumption using a Green Light Optimal Speed Advisory (GLOSA) or eco-driving applications [2,3,4].

Optimizing SPaT information for GLOSA or eco-driving is the main area of concern for this paper. Researchers have shown that using GLOSA significantly reduces fuel consumption. Rakha et al. developed a velocity advisory tool achieving up to a 32% reduction in fuel consumption and 18% reduction in travel time [5]. Chen et al. created a system that can achieve between 8.4% and 17.4% savings in fuel consumption considering grade resistance, communication issues, data errors, and driving latencies [6]. Ahn et al. examined the eco cooperative-adaptive cruise control in connected automated vehicle environment at various levels of market penetration and concluded that a maximum saving of 35.5% at a network level can be achieved at 50% market penetration rate and no congestion [7]. Yang et al. developed an eco-cooperative adaptive cruise control system that can yield as much as 40% savings in fuel consumption [8].

### 1.1. Previous Studies on SPaT Prediction

Several approaches have been used by researchers for predicting SPaT information, mainly the most likely switching times. This includes probabilistic approaches, statistical approaches, and machine learning-based approaches. Among the probabilistic approaches, Bodenheimer et al. used a Bayesian graph-based approach, converting signal states to graphs and computing the probabilities and the most likely timings of transitions [9]. Ibrahim et al. used conditional expectation and confidence-based predictions [10]. Both studies were performed for a single intersection. Among the statistical approaches, Van de Vyvere et al. used historical frequency distributions of SPaT data and used means and medians as predictors, showing that the data 20 min in the past are most relevant [11]. Moghimi et al. developed a sparse multivariate time series statistical model to predict the cycle length along a corridor [12]. Machine learning methods were the most common. Weisheit and Hoyer used support vector machines to decide between three values for switching times in traffic signals [13]. Genser et al. compared linear regression, random forests, and long-short-term memory neural networks (LSTM), and found that LSTM provided the best performance [14]. Eteifa et al. implemented an LSTM-based regression approach and introduced a new loss function for better tradeoffs between short-term and long-term predictions [15]. Islam et al. introduced a convolutional long-short term memory neural network (CNN–LSTM) based architecture and compared it with several machine learning methods, including multilayer perceptrons (MLP), extreme gradient boosting (XGB), gated recurrent units (GRU) and LSTM across two different corridors [16]. The study concluded that CNN–LSTM was the better architecture with a very narrow margin compared to vanilla LSTM.

### 1.2. Problem Statement

While previous studies have considered the application of deep learning models in SPaT predictions, few studies have addressed the specific needs of GLOSA and eco-driving applications. Eco-driving applications require that

1. The prediction is robust to missing data since the percentage of packets decreases both as the distance from the transmitter increases and as the traffic density increases.
2. The prediction is most accurate when the vehicle is within the range of the transmitter: 300 m is the recommended range for DSRC communications and 4G LTE to obtain a reasonable packet delivery rate [1,17]. This distance is equivalent to 19.2 s before the signal changes at a 56 km/h (35 mi/h) speed, which is the speed limit for the Gallows Road Corridor used in this study.
3. The prediction becomes more accurate the closer the time to green is.
4. There is a measure of confidence in the predictions. This way, a vehicle can make a reasonable decision on whether to accelerate or decelerate, considering the level of uncertainty associated with each prediction.

### 1.3. Research Significance and Contribution

This research is a significant step towards enhancing SPaT prediction targeted particularly to the application of GLOSA and eco-driving. The study has the following contributions to the body of knowledge:

1. According to the authors’ knowledge, nobody has used the model architecture introduced in this study, which is based on transformer encoder blocks, in SPaT predictions. The study showed that the predictive power surpasses that of previously used deep learning models.
2. Using an ensemble approach, the study puts forward a novel data-oriented approach for quantifying the certainty in the model predictions. The consensus/variance between the models can be a proxy for confidence in the predictions. This approach is highly extensible and allows for eliminating model hallucinations, which is a major drawback of deep learning models.

The study is based on more than a year’s worth of data from the state of Virginia. No previous studies have tackled this problem in Virginia or used such a lengthy period of data, which captures more temporal variability and covers more of the rare events in traffic.

## 2. Methodology

The major tasks related to SPaT prediction for GLOSA are as follows:

1. Predicting if the phase will change within the next 20 s, because if the phase stays in the same state longer than 20 s, then it does not make a difference to the cars within the communication range of the transmitter.
2. Predicting the exact changing time for phases that would change in less than 20 s in the future.
3. Assigning a level of certainty to the prediction to help the receiving vehicle make a well-informed decision based on the data it receives.

The Section 2.1 and Section 2.2 in the methodology present the data and models used for tasks 1 and 2. The model ensemble section presents the approach and the different hypotheses proposed to address task 3 in this paper.

### 2.1. Data Description

**Figure 1:**
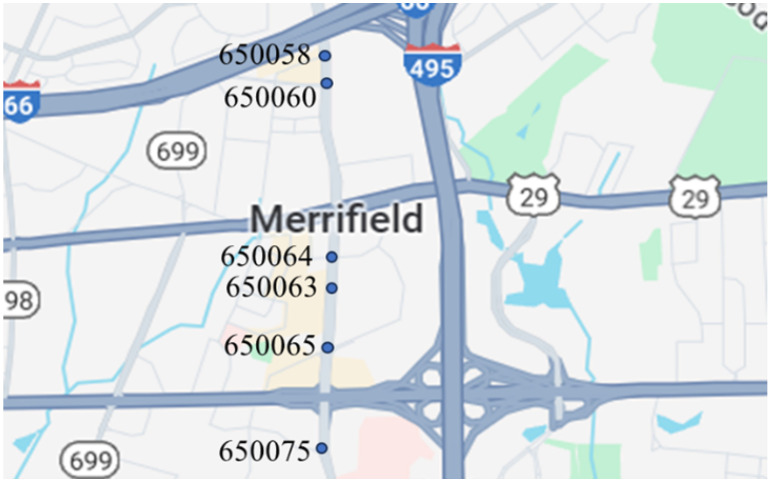
The 6 intersections with Gallows Road in Northern Virginia from which data were collected.

Data were collected from traffic signal controllers of six intersections along Gallows Road in Northern Virginia, as shown in Figure 1. The arterial road connects different land use areas, including residential, commercial, and office buildings. The intersections studied obtain a large amount of traffic throughout the day.

The data stream used includes Signal Phase and Timing (SPaT) information in addition to sensor data. The primary sensors deployed are camera sensors installed atop the traffic signal head mast. These cameras are responsible for detecting the presence or absence of vehicles within predetermined polygons delineated just behind the stop bar for each lane, through variations in pixel intensity. Additionally, pedestrian push buttons are utilized at pedestrian crossing points to capture pedestrian actuation data.

The six signalized intersections cover a wide range of phasing schemes, including four, six, seven, and eight-phase operations, as shown in Table 1. Intersection 650075, which is a T intersection, covers the four-phase operation. Intersections 650063 and 650065 cover a six-phase operation with split phasing for the side road, where each road discharges traffic in all directions in its own phase. Intersection 650058 uses seven phases where the southbound left turn is not allowed. The other two intersections, 650060 and 650064, have all eight phases.

**Figure 2:**
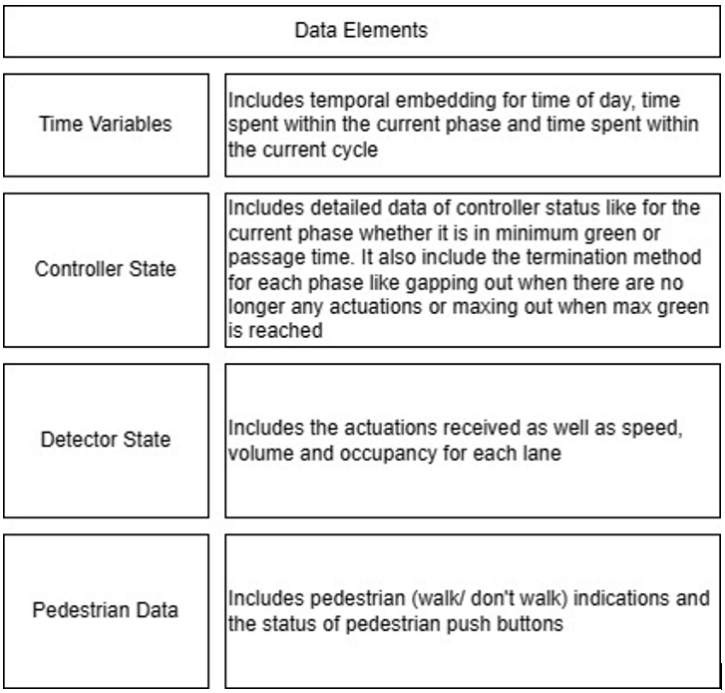
Different variables in SmarterRoads data.

The phasing plans shown in the figure do not fully follow the NEMA phasing scheme and they are shown in the same way dictated by the data. Most notably, the two sides of the barrier phases are swapped. This is because phases 2 and 6 are the ending phases for every cycle. This is mainly to allow for the coordination of the end of the arterial through phases with other traffic signals and to allow for serving these phases for the entire cycle when no actuations are received. The data used were obtained from the Virginia SmarterRoads Web portal. The data were broadcasted by the web API every second and we created a script to capture the data in a JSON file format and construct a database of traffic signal timings. The data were generated by traffic signal controllers and contained in-depth information about the state of the controller. Because of communication losses from the controller to the Web API and then from the API to our servers, the data had missing values that could sum up to 30% for some days. These missing data were coded as missing values and fed to the model as is without imputation to mimic data losses that happen in infrastructure-to-vehicle communication. Figure 2 describes the unique data elements in the VDOT SmarterRoads data.

It should be noted that the level of complexity of SPaT prediction varies significantly across intersections. This variation can be attributed to many factors, including traffic signal controller logic, driver behavior, intersection geometry, and phasing schemas. The intersections examined used D4 traffic signal controllers, and the data used had specific traits that made predictions particularly complex. This includes the following:

1. The sequences of data used have up to 30% missing data. This affects the overall accuracy of the predictions but is more representative of realistic infrastructure to vehicle communication streams.
2. The controller logic includes significant skipping of left turn and side street phases, which makes the predictions highly stochastic, where a single arrival on the secondary road can completely alter the time to green for the primary road.
3. The controller logic for all six intersections includes a setting where, for some cycles, if there are no actuations on any of the approaches, the entire cycle serves phases 2 and 6, which are the primary road through, right and permissive left movements. In this case, the time to green is extended by an entire cycle, which is difficult to predict, especially a long time in advance.

### 2.2. Model Development

Four machine learning models are developed and compared for classifying whether the traffic signal will change within the next 20 s and predicting the precise change time, if less than 20 s. This includes three previously used in the literature, which are MLP, LSTM, and CNN–LSTM. Another architecture based on Transformer Encoder Blocks is proposed, which will be referred to as the Transformer in this paper for brevity despite it not having the decoder element of a full transformer model

#### 2.2.1. Common Elements

All four models used were implemented in the Pytorch deep learning library [18]. All four models were formulated as regression models with the output having *P* features, where *P* is the number of phases being predicted for the intersections. The output features resemble the residual time to green (TTG) or time to red (TTR) in each of the *P* phases of the traffic signal. The models use the Adam optimizer, which was used by all previous deep-learning models predicting SPaT data. Batch size and learning rate were two general hyperparameters that were tested for all models. All models have the same input, which is an F×S input sequence where *F* is the number of features and *S* is the length of the time window chosen to generate the input sequence. After trying multiple values for *S* including 3, 10, 30, 60, and 120 s, respectively, the best results were achieved using a 10 s time window in the past. The Mean Absolute Percentage Error (MAPE) was used as the loss function for all models.

(1) $$\mathrm{MAPE}=\left(\frac{|y_{\mathrm{pred}}-y_{\mathrm{true}}|}{y_{\mathrm{true}}}\right)\times 100$$

MAPE was used as the loss function for all models. The reason for choosing MAPE is that it penalizes errors much more when the prediction is closer to zero. This allows for increased prediction performance when the time to change ground truth is closer to zero. From an application standpoint, the sooner the traffic signal changes, the higher the need for reliable predictions [3]. In a previous study, we analyzed intersection 650065 and showed that the MAPE performs best with short-term predictions, which is the major focus of GLOSA applications [15]. All model hyperparameters were tuned using the Raytune distributed model selection library [19]. The tuning utilizes the Asynchronous Successive Halving (ASHA) algorithm to choose the best hyperparameters that fit the model by employing a selection criterion for aggressive early stopping of less favorable model configurations in favor of more promising configurations to maximize the use of computational resources [20]. Every model type was allowed 8 h of computational time with two Nvidia Tesla A100 GPUs to select the best hyperparameters. The model variants were provided a maximum of 10 epochs for training. The computing node was allowed to train up to 10 model variants in parallel. For each model type, hundreds of configurations were tried to reach the best configurations.

##### MLP

**Figure 3:**
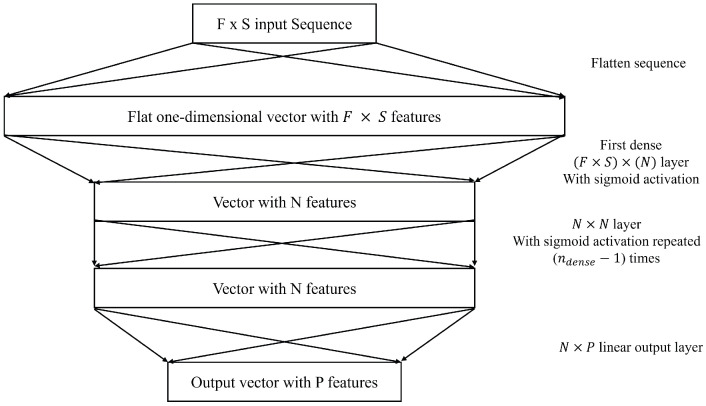
MLP Neural Network Architecture.

MLPs are the simplest baseline neural network architecture that was tested (Figure 3). This multilayer perceptron implementation took the same input as other models, which is the 10 s sequence of data. The features for each one of the 10 time steps were treated as separate features with separate weights assigned to them. In other words, the model uses data from the last 10 s with no context of the order of the data or assigning more value to more recent information. In this case, the model is left on its own to decide the weights to assign to each variable for each time step, which gives the model more control to access very relevant data that occurred further back in the earlier time steps of the input sequence. The model has two main hyperparameters, which are the number of nodes per layer *N* and the number of dense layers with sigmoid activation $n_{dense}$. Other activation functions like ReLu were used and compared with the sigmoid activation function, but the sigmoid activation worked best for this application.

#### 2.2.2. LSTM

**Figure 4:**
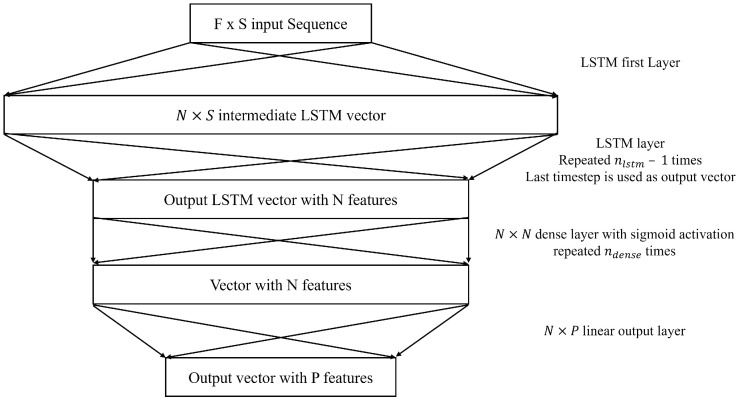
LSTM Neural Network Architecture.

LSTM neural networks have been used in this application multiple times and were one of the best architectures for use with this application [14,15,21]. LSTM acknowledges temporal dependencies between data elements across different time steps. This allows the LSTM neural network to recognize temporal dependencies between signal states and other variables, such as traffic volumes, speeds, vehicle arrivals, and pedestrian arrivals. This facilitates predictions for the network, considering both short-term dependencies and long-term trends within the data. The structure of LSTM used in this study (Figure 4) is the one proposed by Graves in 2013 [22]. The LSTM cell can be described using the following five equations:

(2) $$\begin{array}{c} i_{t}=\sigma \left(W_{xi}x_{t}+W_{hi}h_{t-1}+W_{ci}c_{t-1}+b_{i}\right) \end{array}$$

(3) $$\begin{array}{c} f_{t}=\sigma \left(W_{xf}x_{t}+W_{hf}h_{t-1}+W_{cf}c_{t-1}+b_{f}\right) \end{array}$$

(4) $$\begin{array}{c} c_{t}=f_{t}c_{t-1}+i_{t}\tanh\left(W_{xc}x_{t}+W_{hc}h_{t-1}+b_{c}\right) \end{array}$$

(5) $$\begin{array}{c} o_{t}=\sigma \left(W_{xo}x_{t}+W_{ho}h_{t-1}+W_{co}c_{t-1}+b_{o}\right) \end{array}$$

(6) $$\begin{array}{c} h_{t}=o_{t}\tanh\left(c_{t}\right) \end{array}$$

The variable $x_{t}$ refers to the input at time *t*. Input, output, and forget gates are referred to as *i*, *o*, and *f*, respectively. The inner LSTM cell is referred to as *c*, which is where information is stored. The variable *h* refers to the hidden state. Weights are referred to as *W* and biases as *b*. The Tanh activation is used for the LSTM layers. In this model, there are a total of three main hyperparameters that were tuned. *N* was used as the number of features of the LSTM sequence, the number of features in the LSTM output vector, and the number of neurons in the dense layer. The variable $n_{lstm}$ is the number of LSTM layers. The variable $n_{dense}$ is the number of dense layers with sigmoid activation used to aggregate the LSTM output.

#### 2.2.3. CNN–LSTM

**Figure 5:**
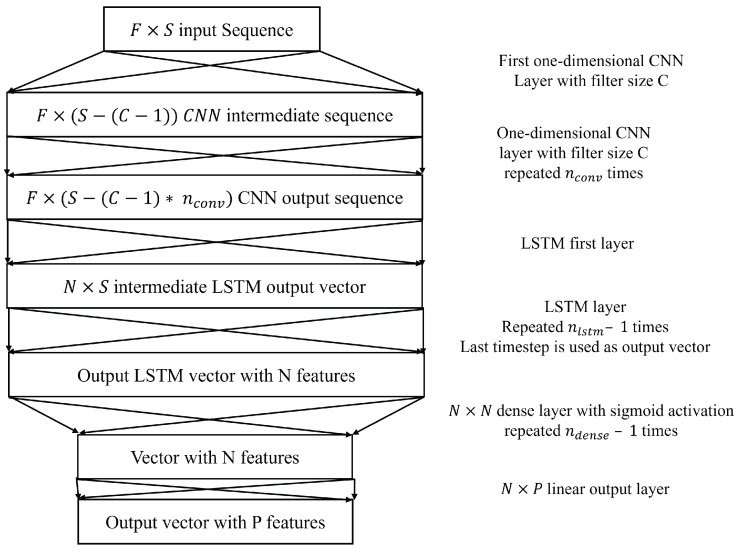
CNN LSTM Neural Network Architecture.

CNN–LSTM was proposed in a previous study as the best method for SPaT prediction, albeit being very similar in prediction performance to a simple LSTM [16]. Using two-dimensional convolutions, however, was found to not be very useful to aggregate features because, unlike computer vision applications where features are spatially correlated, the controller features are not spatially correlated, so the output of the two-dimensional CNN–LSTM would be highly reliant on the exact order of the chosen features. For this reason, the one-dimensional CNN–LSTM is better suited for this specific problem, where the convolutions can detect specific trends observed across each feature separately and feed that into the LSTM to capture temporal dependencies. The CNN–LSTM has five different hyperparameters, which include the filter size C for the convolution, the number of convolution layers $n_{conv}$, the number of LSTM layers $n_{lstm}$, the number of dense layers $n_{dense}$ and the number of neurons in each layer N. N refers to the number of LSTM cells and the number of neurons for the dense layer. The CNN–LSTM structure is shown in Figure 5.

#### 2.2.4. Transformer

**Figure 6:**
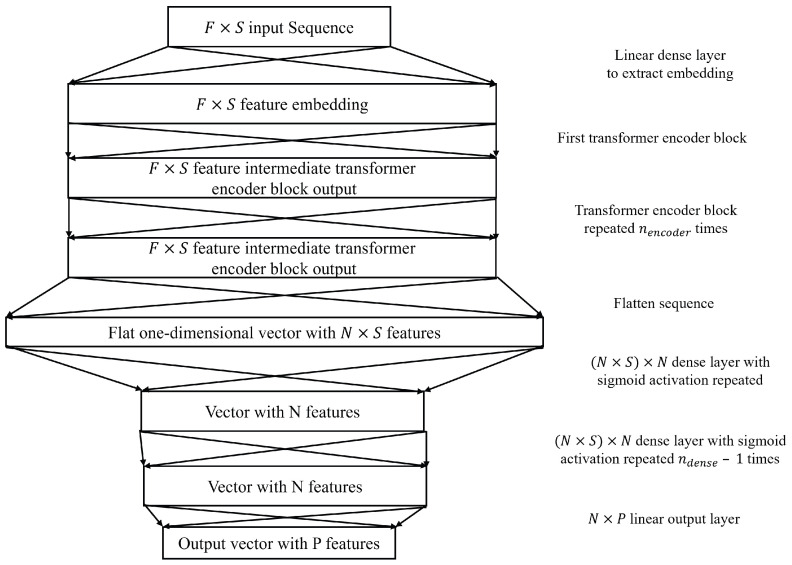
Transformer encoder-based architecture.

A typical transformer architecture comprises an encoder block to process the inputs and apply self-attention between the input sequence time steps and a decoder block to conduct the same for the output, such that the mapping between the input and output sequences can be established. The architecture proposed in Figure 6 uses only the encoder block of the transformer and replaces the decoder block with dense layers using sigmoid activations and a final linear layer for regression. The encoder’s primary function is to capture temporal dependencies within the input data, which is essential for SPaT prediction.

Since the tasks involve classification and regression rather than sequence generation, the inclusion of a decoder was deemed unnecessarily complex, with no clear benefits to performance. An alternative approach was explored by framing the problem as a sequence-to-sequence task, where traffic signal states were represented as binary sequences (1 for green, 0 for red), but no significant improvements were observed. Additionally, the use of a decoder block before the final classification/regression layer did not enhance model performance. Consequently, a simpler and more effective architecture was adopted, utilizing a linear layer to map the encoder’s representation to the output. The encoder block structure is similar to that described by Vaswani et al. [23].

**Figure 7:**
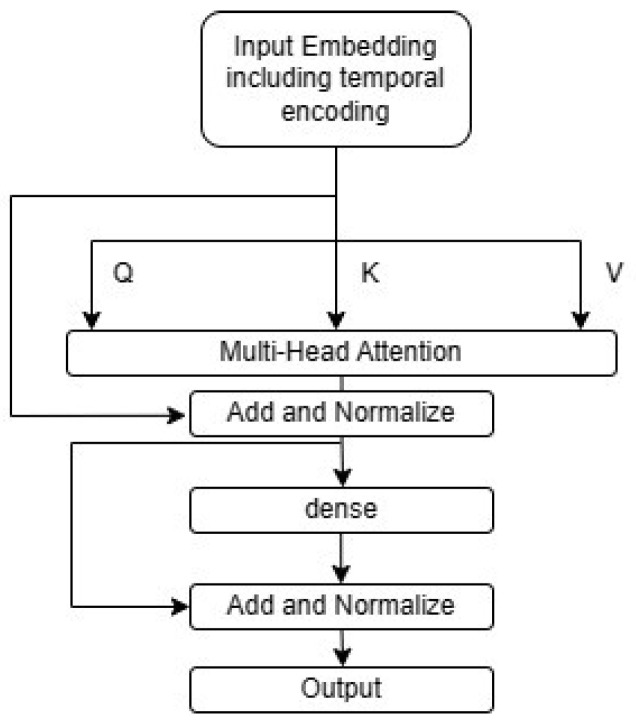
Encoder block architecture.

The main model-specific hyperparameters in this case include *N*, which is the size of the input embedding and also the size of the transformer output and the size of the dense layers prior to the output; $n_{head}$, which is the number of attention heads in each encoder multi-headed attention; $n_{encoder}$, which is the number of encoders stacked in the model and $n_{dense}$, which is the number of dense layers with sigmoid activations to aggregate the transformer encoders’ output before the output linear layer. The transformer encoder block architecture is shown in Figure 7, which is adapted from Viswani et al. [23].

The variables *Q*, *K*, and *V* in this case represent the query, key, and value, which are linear projections of the input embedding that includes the temporal encoding for each attention head. For each head, the dot product attention can be computed as follows [23]:

(7) $$\mathrm{Attention}\left(Q,K,V\right)=\mathrm{softmax}\left(\frac{QK^{T}}{\sqrt{d_{k}}}\right)V$$

where the matrix multiplication between *Q* and *K* is a measure of similarity between the query and key matrices. This is divided by a scaling factor equal to the square root of the dimension of the key matrix $d_{k}$ for normalization. The purpose of this dot product attention between *Q* and *K*, which are linear projections of the input sequence, is self-attention. The dot product is a similarity metric between *Q* and *K*, which allows the model to distinguish which time steps are more relevant to the prediction of time to change and which are less relevant. Finally, this is subjected to the softmax function to magnify the differences between self-attention weights to help the model decide which time steps are most relevant to the prediction. This is then multiplied by the value matrix to obtain the output of the dot product attention, which has all the features with a special focus on the timesteps that are most relevant to the prediction.

### 2.3. Ensemble Approach

The four models MLP, LSTM, CNN–LSTM, and Transformer vary significantly in how they handle data and the computations they require to make a prediction. We can make use of this variation in model logic in making an ensemble-based prediction. One of the main prerequisites for a successful ensemble model is having diversity in the ensemble constituent models [24]. This is achieved in two ways:

1. By using four different architectures, the data are subjected to different processes and logic in order to extract the relevant features and make the prediction. This variance in logic, as represented by the different model architectures, adds to the logical diversity of the model.
2. For each of the four models, during hyperparameter tuning we generate multiple variants where the number of parameters used to express the model for each variant varies significantly. This means that the variation in model size and number of parameters, even for the same model architecture, allows for diversity in the complexity of the constituent models for the ensemble.

For the first task—predicting whether the traffic signal will change within the next 20 s—the ensemble model is evaluated using three different aggregation methods: (1) calculating the mean of the individual model predictions, (2) computing the median of the predictions, and (3) implementing a voting mechanism based on whether the predicted change occurs within the next 20 s.

For the second task—predicting the precise time of the signal change within a 20 s window—the ensemble model is assessed using two combination strategies: taking the mean and the median of the individual model predictions.

Using the ensemble approach for the prediction of SPaT information is hypothesized to have two key sources of value:

**Hypothesis** **1.** *Using multiple diverse predictions instead of one is likely to improve prediction performance by eliminating outlier predictions, which are also known as ’model hallucinations’. The constituent models of the ensemble are unlikely to provide the same incorrect prediction, whether that error is because of bias or variance, unless the bias or variance is inherent to the data and has nothing to do with the model.*

**Hypothesis** **2.** *For each prediction, reporting consensus between the constituent models of the ensemble can serve as a powerful indicator of confidence in the prediction. This means that predictions agreed upon by more models are more likely to be closer to the true value. This can be easily tested by looking at the error distribution of different predictions and how it varies with consensus between the models. The expected behavior is that as consensus increases, the mean error of the predictions should decrease, and the percentage of distant outlier predictions should decrease.*

## 3. Results and Analysis

This section is subdivided into three subsections, presenting the results for the three tasks outlined at the beginning of the methodology. This helps us better understand how well the models perform on each task and how that affects the overall performance of the ensemble prediction. Each of the three tasks is carried out using the top three ranked models of each architecture, totaling 12 different models. The models are ranked based on the Mean Absolute Percentage Error (MAPE). Table 2, Table 3, Table 4 and Table 5 provide the hyperparameter configuration of the top-ranked 12 models for each of the six intersections.

Table 2, Table 3, Table 4 and Table 5 show that for each model and each intersection, there is a variation between the top three chosen models in terms of the hyperparameters. This variation leads to the diversity of the top three selected models, which is what allows the top three models to be useful for the model ensemble.

### 3.1. Task 1: Identifying Change Times Less than 20 s in the Future

The task aims to distinguish between predictions less than 20 s ahead and those further in the future. Each of the 12 models makes a regression prediction, which is then converted to a binary outcome: 1 if under 20 s, 0 if over. Regression models are used for their superior performance in classification due to their nuanced loss function. Three ensemble techniques are tested: mean, median, and majority vote of the 12 models. The table shows the classification accuracy for each model at each intersection and the ensemble methods.

Table 6 shows that all architectures achieve accuracies around 95%. The transformer architecture outperforms others at all intersections except for 650075, where the MLP slightly outperforms the transformer. The MLP is the second-best model, demonstrating that treating sequence time steps as separate features is effective. LSTM is the second worst, and CNN–LSTM is the worst. The best overall performance is achieved by taking the mean of the 12 models compared to the 20 s cutoff, resulting in the highest accuracy at all intersections except 650058 and 650064, where it is slightly outperformed by the best transformer model. Median and voting strategies are outperformed by the best transformer model for all intersections.

### 3.2. Task 2: Predicting the Exact Time to Change When Less than or Equal to 20 s

After deciding whether the time to change for each phase is within 20 s in the future. The next task is to predict the exact time to change for each phase. Table 7 and Table 8 show the MAPE and mean absolute error for each model for the subset of data with a time-to-change less than 20 s in the future for all six intersections. The prediction performance of the mean and median of the model ensemble is also provided in both tables.

Table 7 and Table 8 both show that the proposed transformer-encoder-based architecture outperforms all the other architectures for all intersections except for intersection 650060, where CNN–LSTM outperforms the transformer architecture. Aside from intersection 650060, CNN–LSTM is slightly outperformed by MLP. Vanilla LSTM is the worst-performing by a small margin. All architectures have a reasonably low MAPE between 11 and 23%, meaning that the absolute error is between 11 and 23% of the predicted value. This results in the best models being off by 0.96 s from the true value on average and the worst are about 2.05 s from the true value on average, as shown in Table 8. The ensemble shows that taking the mean or the median value of all models enhances the prediction. The median value, however, is the best estimator for the prediction because it is robust to outliers, unlike the mean. The median value always leads to a non-trivial improvement over the best model prediction across all 12 models. This proves Hypothesis 1, outlined at the end of the methodology section. By taking the median of the predictions of 12 diverse models, the hallucinations of a subset of the models can be rectified, leading to an overall better prediction performance and a result that outperforms every individual model.

### 3.3. Task 3: Assigning a Level of Certainty to the Prediction

The third task, which is the most important and the least addressed in the literature, is to assign a level of certainty to each prediction. For each input and phase, the 12 best models provide a prediction. Due to model errors from bias and variance, models are unlikely to converge on the same incorrect prediction unless there is an inherent data error.

A certainty metric is defined based on model consensus. Given a set of predictions *P* for the same input with a median prediction *m*, an adjustable tolerance level *t* (typically within 20%) is used. The number of predictions within this tolerance indicates the certainty level. Certainty is computed by the number or percentage of agreeing models. The number of models in consensus is preferred over the percentage, as the latter could be misinterpreted as the probability of a correct prediction.

(8) certainty=count(p)∀p∈P,(1−t)m<p<(1+t)m

**Figure 8:**
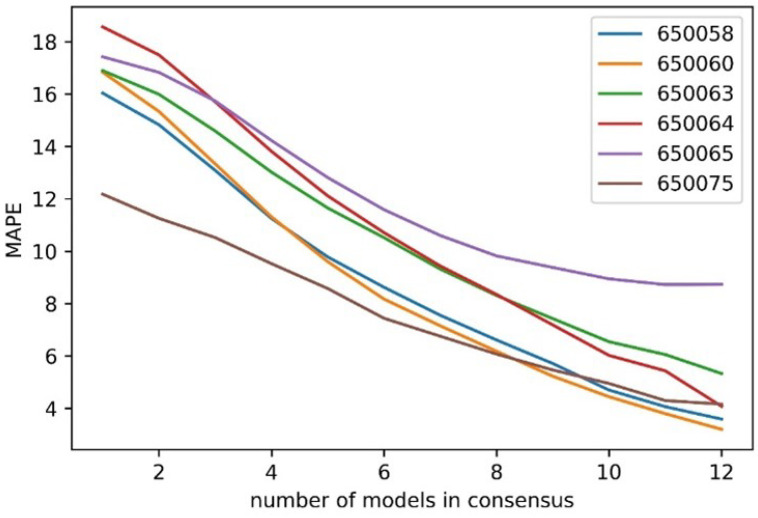
MAPE as a function of the level of consensus with a 5% tolerance level for all 6 intersections.

Testing this formulation, a tolerance level of 5% is chosen and the count of the models within this tolerance level is evaluated. This results in 12 different levels of confidence from 1 to 12, where 1 indicates the least confidence (a minimum of 1 model agrees with this median within tolerance) and 12 indicates the highest confidence (all 12 models agree on this median within tolerance). The MAPE levels for each level of consensus are shown in Figure 8. It should be noted that for each level of consensus, the error shown is for this level of consensus or higher, so a level of consensus of 1 shows the overall MAPE as the entire dataset has a minimum level of consensus of 1 model. 

**Figure 9:**
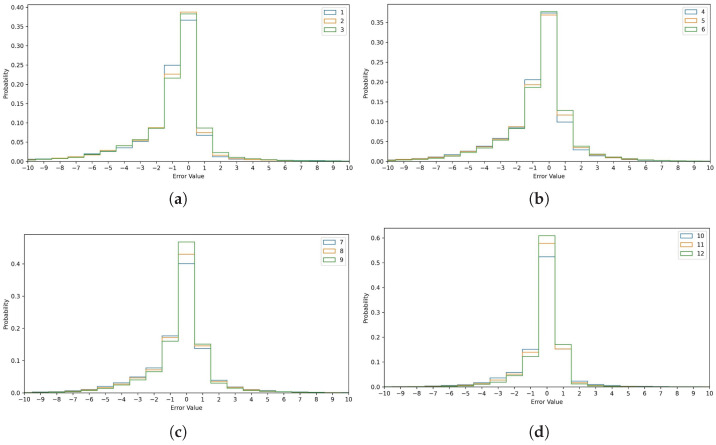
The error distributions (**a**) consensus values 1 to 3. (**b**) consensus values 4 to 6. (**c**) consensus values 7 to 9. (**d**) consensus values 10 to 12.

Figure 8 highlights the effectiveness of the consensus metric in distinguishing high-confidence from low-confidence predictions. Intersection 650058 has a 16.04% MAPE overall, which drops to 3.59% when all 12 models reach a consensus. At intersection 650060, the MAPE decreases by 80%, from 16.83% to 3.2%, for the highest consensus score. For intersection 650063, the MAPE falls from 16.89% to 5.33% with a consensus score of 12, confirming Hypothesis 2 from the methodology section. The consensus score quantifies the prediction uncertainty before the true value is known. This approach is adaptable to all model types, including statistical and machine learning models, and can be combined with other certainty measures like probabilistic predictions. Figure 9 shows the error probability distribution for each consensus level from 1 to 12. The error is computed using the equation:

(9) $$\mathrm{Error}=y_{\mathrm{pred}}-y_{\mathrm{true}}$$

where $y_{pred}$ is the median of the model-predicted values and $y_{true}$ is the ground truth. This means that a positive error refers to an overestimation and a negative error refers to an underestimation of the traffic signal time to change. The error distribution shown in Figure 9 shows errors between −10 s and +10 s for the 20 s prediction window. Errors greater than 10 s are distant outliers and their percentages for each level of consensus are shown in Table 8 for completeness. Errors are aggregated across all six intersections. The predictions are all rounded to the nearest second, and the ground truth is in seconds. This means that the values shown for each bar in the histograms in Figure 9 are the exact values of the error. For example, the middle bar represents the percentage of the time where the error is zero, so the rounded prediction is exactly equal to the ground truth.

**Figure 10:**
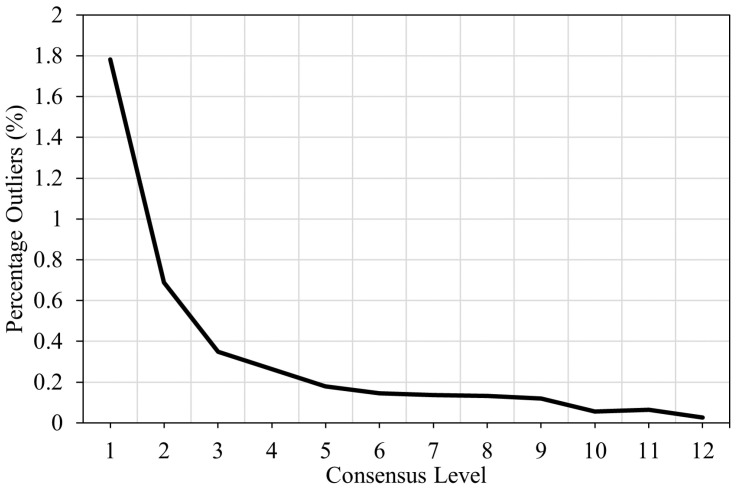
Percentage of outlier predictions with an error greater than 10 s.

Figure 9 and Figure 10 illustrate the error distributions for different consensus levels at a 5% tolerance. Lower consensus levels have flatter error curves. At a consensus level of 1, the median prediction has zero error 36% of the time, while at level 12, it has zero error 61% of the time. The error is within 1 s 90.2% of the time at level 12 but only 68.4% at level 1. The ensemble’s error exceeds 10 s 1.782% of the time at level 1, compared to just 0.026% at level 12. Thus, at level 1, the ensemble is 68.5 times more likely to be off by more than 10 s than at level 12. This strongly supports Hypothesis 2, showing that the consensus level is a reliable indicator of prediction accuracy.

### 3.4. Fuel Consumption

**Figure 11:**
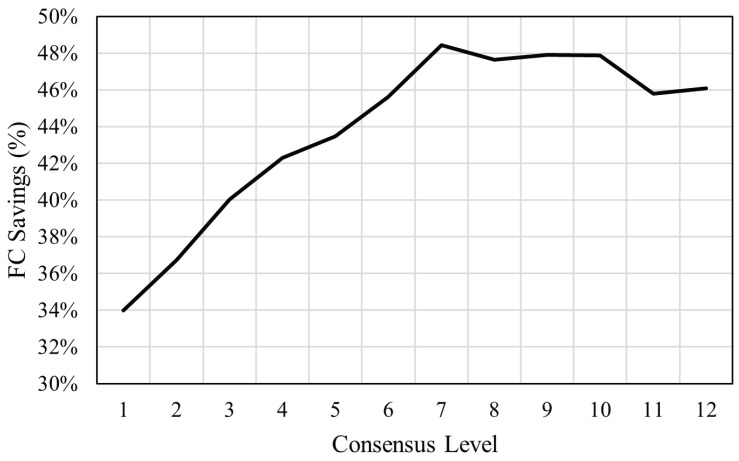
Effect of level of consensus on fuel savings.

To further quantify the effect of the consensus level on the overall performance of the GLOSA system, the effect on a single vehicle going through an intersection is simulated using the stochastic control algorithm developed by Shafik et al. [3]. The effect of the level of consensus on fuel consumption is shown in Figure 11.

Figure 11 shows that if more than half the models agree on the prediction (level of consensus of 7 or more), the maximum level of fuel savings of about 48% can be achieved relative to then uninformed driver behavior obtained from field data from Chen et al. [6]. The fuel savings continue to decrease as the level of consensus of the model decreases, with the lowest level of consensus achieving approximately a 34% fuel saving. The ensemble model achieves an overall average fuel consumption saving of approximately 42%, which is comparable to the savings obtained using the best-performing transformer prediction model. However, incorporating the consensus score can further enhance fuel savings, reaching up to 48%, while also providing a reliable confidence metric for assessing prediction performance.

### 3.5. Considerations for Field Deployment of Model Ensembles

Deploying machine learning model ensembles for real-time traffic signal prediction presents several challenges and practical considerations that must be addressed to ensure reliable performance in real-world environments. These considerations include computational efficiency, data reliability, and communication latency. While deep learning models, including transformer-based architectures, can be computationally intensive, the models used in this study are relatively lightweight and efficient, making them suitable for deployment in traffic infrastructure. With the increasing prevalence of large-scale language models operating on low-power devices, similar lightweight models can be efficiently deployed on edge computing systems such as roadside units or in-vehicle processors. Additionally, in highly resource-constrained environments, endpoints with limited processing power can be connected to cloud-based or edge computing resources that handle predictions and transmit the results in real time.

#### 3.5.1. Data Reliability and Handling Missing Data

A key concern in real-world deployment is data reliability, particularly in environments where missing or incomplete data are common. The dataset used in this study originates from high-fidelity traffic signal controllers, which inherently capture real-world issues such as communication disruptions and data losses. By training models on imperfect datasets, the proposed approach is more resilient to such challenges.

#### 3.5.2. Communication Latency and Prediction Timing

In real-time applications, communication latency can impact the effectiveness of traffic signal predictions. For our model ensemble, the prediction time was always under 500 ms (mostly under 300 ms), which allows for up to another 500 ms of communication delay for a prediction made one second in advance. Predictions made approximately one second before transmission ensure that the information remains relevant when received by vehicles or infrastructure. Additionally, predictions should be communicated as future timestamps of signal changes rather than time interval durations. This approach minimizes the risk of misinterpretation due to communication outages and ensures that predictions remain actionable for eco-driving and traffic safety applications.

## 4. Conclusions and Recommendation

This study proposed a transformer encoder-based architecture to enhance SPaT data through better predictions of traffic signal switch times and provide levels of confidence in these predictions for use in GLOSA applications. The transformer was compared against other architectures previously used in the literature, which include MLP, LSTM, and CNN–LSTM. The transformer was found to outperform the other three architectures in two different tasks related to GLOSA. These predict whether a specific phase would change within the next 20 s and predict the exact changing time within that period. Three variants of each model type were integrated into an extensible ensemble framework. The ensemble was proven to improve the overall prediction performance to be better than the best model performance by reducing model hallucinations by taking the mean of all models to identify a change time within 20 s and the median for prediction of the actual switch time within the 20 s window. The consensus of models is a good indicator of the quality and the certainty of predictions. The highest confidence predictions had as much as an 80% reduction in MAPE compared to the lowest confidence prediction. The level of consensus can provide a much-needed estimation of the confidence in the most likely switching time in the SPaT messages. Future areas of study might examine the field-testing of ensemble predictions and consensus-based certainty in a transportation network. Issues such as communication latencies, interference, queueing and vehicle dynamics can affect the degree to which the vehicles can benefit from enhanced SPaT messages. Quantifying the error in the SPaT predictions opens the door for more nuanced error-aware eco-driving that considers the certainty level in the prediction and balances the risks and rewards associated with following the SPaT predictions.

## Figures and Tables

**Table 1:** Phasing schemes for all 6 intersections studied.

Intersection	Ring Barrier Diagram	Intersection	Ring Barrier Diagram
650058	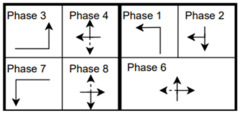	650064	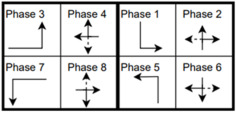
650060	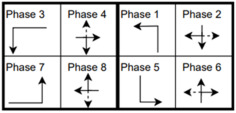	650065	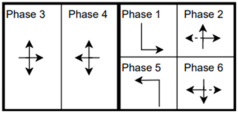
650063	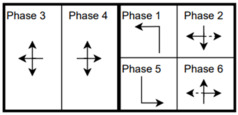	650075	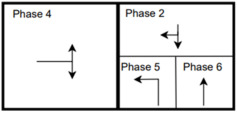

**Table 2:** MLP best model configurations.

Intersection	Model Rank	lr	Batch Size	Neurons Per Layer	n Layers
650058	1	0.00019	16	300	2
	2	0.00029	16	240	2
	3	0.00042	32	240	2
650060	1	0.00062	64	300	2
	2	0.00046	32	270	2
	3	0.00026	16	240	2
650063	1	0.00025	32	210	2
	2	0.00035	16	150	2
	3	0.00092	128	240	4
650064	1	0.00025	16	210	2
	2	0.00019	16	300	2
	3	0.00046	32	270	2
650065	1	0.00041	32	270	2
	2	0.00045	64	210	2
	3	0.00019	16	240	2
650075	1	0.00032	16	150	2
	2	0.00040	16	210	1
	3	0.00105	64	180	2

**Table 3:** LSTM best model configurations.

Intersection	Model Rank	lr	Batch Size	Neurons Per Layer	n Layers Lstm	n Layers Dense
650058	1	0.00134	128	300	2	1
	2	0.00135	128	210	2	1
	3	0.00068	32	120	2	1
650060	1	0.00111	32	180	2	1
	2	0.00134	128	300	2	1
	3	0.00168	64	180	2	1
650063	1	0.00024	16	180	2	2
	2	0.00040	32	180	2	1
	3	0.00068	32	120	2	1
650064	1	0.00068	32	120	2	1
	2	0.00119	32	120	2	2
	3	0.00120	32	150	1	1
650065	1	0.00040	32	180	2	1
	2	0.00139	32	150	2	1
	3	0.00068	32	120	2	1
650075	1	0.00034	16	90	2	2
	2	0.00111	32	180	2	1
	3	0.00068	32	120	2	1

**Table 4:** CNNLSTM best model configurations.

Intersection	Model Rank	lr	Batch Size	Neurons Per Layer	n Layers Lstm	n Layers Dense
650058	1	0.00040	16	300	2	1
	2	0.00101	32	240	2	1
	3	0.00109	64	240	2	1
650060	1	0.00080	32	300	4	1
	2	0.00021	16	300	4	1
	3	0.00040	16	300	2	1
650063	1	0.00109	64	240	2	1
	2	0.00061	16	120	4	1
	3	0.00048	32	300	2	1
650064	1	0.00253	128	240	3	1
	2	0.00050	16	120	3	1
	3	0.00230	128	210	4	1
650065	1	0.00041	16	240	4	1
	2	0.00121	128	150	4	1
	3	0.00050	16	120	3	1
650075	1	0.00041	16	240	4	1
	2	0.00080	32	300	4	1
	3	0.00168	64	120	2	1

**Table 5:** Transformer best model configurations.

Intersection	Model Rank	lr	Batch Size	Embed Dim (N)	n Heads	n Encoder Layers	n Layers Dense
650058	1	0.00020	32	300	1	2	2
	2	0.00016	32	240	3	1	4
	3	0.00014	32	210	2	2	2
650060	1	0.00018	16	60	3	2	2
	2	0.00046	64	90	3	2	4
	3	0.00042	32	60	5	2	2
650063	1	0.00067	64	30	5	4	2
	2	0.00080	128	60	5	1	2
	3	0.00018	16	60	3	2	2
650064	1	0.00014	32	210	2	2	2
	2	0.00020	32	300	1	2	2
	3	0.00046	64	120	1	1	2
650065	1	0.00075	128	150	1	1	2
	2	0.00041	128	120	3	1	2
	3	0.00024	32	210	1	1	4
650075	1	0.00029	32	120	1	1	4
	2	0.00020	32	300	1	2	2
	3	0.00018	16	60	3	2	2

**Table 6:** Classification accuracy of different models distinguishing whether a signal phase will change 20 s in the future.

Intersection	Model Rank	LSTM	MLP	CNN-LSTM	Trans-Former	Mean	Median	Vote
650058	1	94.84%	94.97%	94.37%	95.31%	95.24%	95.12%	95.08%
	2	94.59%	94.71%	94.63%	95.25%			
	3	94.68%	94.82%	94.64%	95.14%			
650060	1	95.20%	95.23%	93.47%	95.04%	95.53%	95.03%	95.17%
	2	94.40%	95.11%	93.22%	95.09%			
	3	94.20%	95.05%	93.58%	95.32%			
650063	1	95.95%	95.90%	95.88%	96.07%	96.17%	96.10%	96.08%
	2	95.70%	96.01%	95.54%	95.96%			
	3	95.87%	95.84%	95.91%	96.04%			
650064	1	94.98%	95.84%	94.20%	96.59%	96.51%	96.04%	96.17%
	2	95.06%	95.81%	93.77%	96.24%			
	3	95.67%	95.97%	94.36%	96.45%			
650065	1	95.63%	96.36%	94.33%	96.39%	96.43%	96.08%	96.07%
	2	95.88%	95.75%	94.18%	96.35%			
	3	95.76%	95.92%	94.39%	96.08%			
650075	1	96.01%	96.63%	96.01%	96.07%	96.73%	96.07%	95.86%
	2	96.08%	96.19%	95.54%	96.18%			
	3	95.77%	96.05%	95.59%	96.04%			

**Table 7:** Mean Absolute Percentage Error of Each Model for Values under 20 s.

Inter-Section	Model Rank	LSTM	MLP	CNN- LSTM	Trans-Former	Mean	Median
650058	1	20.45	16.65	19.40	15.39	15.58	15.03
	2	19.70	16.33	20.43	15.95		
	3	21.15	16.68	20.90	15.29		
650060	1	22.64	19.49	17.80	18.07	16.72	15.89
	2	20.90	19.12	17.84	19.01		
	3	21.79	19.29	20.27	19.03		
650063	1	22.47	19.65	20.62	16.29	16.45	15.61
	2	23.14	19.20	18.67	16.90		
	3	22.47	19.19	21.23	17.56		
650064	1	22.29	21.28	21.92	19.88	18.31	17.60
	2	23.08	21.18	22.43	18.75		
	3	25.07	22.58	23.09	19.38		
650065	1	22.74	19.97	19.65	17.56	16.70	16.32
	2	22.21	18.97	19.82	17.90		
	3	22.54	19.19	20.33	17.34		
650075	1	16.38	15.01	12.95	11.37	11.94	10.99
	2	17.64	13.68	14.96	13.25		
	3	17.62	13.64	16.25	12.94		

**Table 8:** Mean Absolute Error of Each Model for Values under 20 s.

Inter-Section	Model Rank	LSTM	MLP	CNN- LSTM	Trans-Former	Mean	Median
650058	1	1.585	1.456	1.572	1.345	1.330	1.309
	2	1.509	1.450	1.707	1.428		
	3	1.636	1.480	1.614	1.351		
650060	1	1.839	1.720	1.516	1.684	1.483	1.419
	2	1.619	1.728	1.501	1.760		
	3	1.677	1.773	1.621	1.718		
650063	1	1.785	1.647	1.711	1.473	1.446	1.428
	2	1.786	1.695	1.632	1.511		
	3	1.776	1.662	1.702	1.573		
650064	1	1.843	1.909	1.864	1.801	1.644	1.615
	2	1.945	1.898	1.917	1.685		
	3	2.051	2.052	1.954	1.782		
650065	1	1.836	1.659	1.776	1.605	1.502	1.496
	2	1.757	1.688	1.811	1.595		
	3	1.821	1.671	1.762	1.556		
650075	1	1.177	1.194	1.033	0.962	0.933	0.888
	2	1.264	1.086	1.247	1.056		
	3	1.213	1.074	1.168	1.005		

## Data Availability

The datasets presented in this article are not readily available because they are owned by the Virginia Department of Transportation (VDOT). Requests to access the datasets should be directed to VDOT.

## Abbreviations

The following abbreviations are used in this manuscript:

TSC	Traffic Signal Control
SPaT	Signal phase and timing
GLOSA	Green light optimal speed advisory
MLP	Multilayer perceptrons
LSTM	long-short-term memory neural networks
CNNLSTM	Convolutional long-short term memory neural networks
I2V	infrastructure to vehicle
ITS	intelligent transportation systems
DSRC	Dedicated short-range communications
LTE	Long-Term Evolution
MAPE	Mean Absolute Percentage Error
ASHA	Asynchronous Successive Halving
GPU	Graphics processing unit
NEMA	National Electrical Manufacturers Association
TTG	Time to green
TTR	Time to red
XGB	extreme gradient boosting
GRU	gated recurrent units
